# Effects of Age in Fecal Microbiota and Correlations with Blood Parameters in Genetic Nucleus of Cattle

**DOI:** 10.3390/microorganisms12071331

**Published:** 2024-06-29

**Authors:** Richard Estrada, Yolanda Romero, Deyanira Figueroa, Pedro Coila, Renán Dilton Hañari-Quispe, Mery Aliaga, Walter Galindo, Wigoberto Alvarado, David Casanova, Carlos Quilcate

**Affiliations:** 1 Dirección de Desarrollo Tecnológico Agrario, Instituto Nacional de Innovación Agraria (INIA), Lima 15024, Peru; yolanda.bioinfo@gmail.com (Y.R.); deyanirafigueroa66@gmail.com (D.F.); dcasanova@inia.gob.pe (D.C.); 2 Facultad de Medicina Veterinaria y Zootecnia, Universidad Nacional del Altiplano, Puno 21001, Peru; pcoila@unap.edu.pe (P.C.); rhanari@unap.edu.pe (R.D.H.-Q.); mlaliaga@unap.edu.pe (M.A.); waltergalindos@gmail.com (W.G.); 3 Facultad de Ingeniería Zootecnista, Agronegocios y Biotecnología, Universidad Nacional Toribio Rodríguez de Mendoza de Amazonas (UNTRM), Chachapoyas 01001, Peru; wigoberto.alvarado@untrm.edu.pe

**Keywords:** cattle fecal microbiota, age-related changes, 16S rRNA gene sequencing, blood parameters, functional diversity

## Abstract

This study aimed to determine the impact of age on the fecal microbiota in the genetic nucleus of cattle, with a focus on microbial richness, composition, functional diversity, and correlations with blood parameters. Fecal and blood samples from 21 cattle were analyzed using 16S rRNA gene sequencing. Older cattle exhibited greater bacterial diversity and abundance, with significant changes in alpha diversity indices (*p* < 0.05). Beta diversity analysis revealed significant variations in microbial composition between age groups and the interaction of age and sex (*p* < 0.05). Correlations between alpha diversity, community composition, and hematological values highlighted the influence of microbiota on bovine health. Beneficial butyrate-producing bacteria, such as *Ruminococcaceae*, were more abundant in older cattle, suggesting a role in gut health. Functional diversity analysis indicated that younger cattle had significantly more abundant metabolic pathways in fermentation and anaerobic chemoheterotrophy. These findings suggest management strategies including tailored probiotic therapies, dietary adjustments, and targeted health monitoring to enhance livestock health and performance. Further research should include comprehensive metabolic analyses to better correlate microbiota changes with age-related variations, enhancing understanding of the complex interactions between microbiota, age, and reproductive status.

## 1. Introduction

The intestinal microbiota is closely associated with various physiological functions of the host and plays a crucial role in the health and performance of livestock. This complex system, involved in essential functions such as nutrient absorption, local immunity, and overall metabolic health, is influenced by environmental and host factors such as diet, genetics, and age, and has significant implications for animal health and development [1,2,3,4].

The microbiota of ruminants significantly influenced by age impacts the health and metabolic processes of these animals [5]. Young cattle have a diverse microbiota that becomes more complex with maturity, which is crucial for efficient digestion and general health [6]. In lambs, for example, the rumen microbiota undergoes significant changes with age, such as an increase in the diversity and abundance of certain beneficial bacterial species that enhance fermentation and nutrient absorption [7]. Understanding this dynamic is vital to improve dietary and management practices, thereby optimizing the growth and health of animals. As animals mature, the composition of the microbiota shifts, with increases in fiber-digesting bacteria that improve the nutritional capacity of the host. Furthermore, age-related changes in the microbiota are connected to various blood parameters, such as glucose and cholesterol levels, reflecting metabolic adjustments during animal maturation [8].

Ruminant stomach bacteria decompose cellulose, hemicellulose, and other nutrients like sugars, fats, and proteins [9]. The gut microbiota of cattle consists of diverse bacterial phyla, including *Firmicutes*, *Bacteroidetes*, *Proteobacteria*, and *Actinobacteria*, each of which plays a vital role in intestinal health [10,11]. Understanding these phyla helps elucidate the dynamics of the gut microbiota, guiding the development of probiotics and diets to improve bovine health and productivity [10].

Simmental cattle are valuable for both meat and milk production, exhibiting rapid growth and efficient feed conversion [12]. Recent genomic studies have identified variants associated with meat quality, including genes related to growth rate and muscle development [13,14]. Additionally, Simmental cows demonstrate good milk production capacity, making them valuable for dual-purpose farming [15,16]. Studying the intestinal microbiota is crucial because it plays a significant role in nutrient absorption, immune system modulation, and overall health maintenance [17]. The gut microbiota changes with age, affecting metabolic, immune, and cognitive health. Probiotics can modulate the microbiota, promoting healthy aging by improving intestinal barrier function and nutrient absorption, especially in aged animals [18]. Early supplementation with probiotics benefits young animals, such as newborn calves, by improving their growth, intestinal health, and disease resistance [19]. This knowledge can be used to develop targeted probiotic therapies that enhance beneficial microbial populations and improve gut health [20].

Additionally, disruptions in the gut microbiota have been linked to various diseases, highlighting the importance of understanding its composition and functions to create effective probiotic interventions [21]. Studying the microbiota from a genetic core provides information on its hereditary influence on health, allowing the development of personalized probiotic therapies for different animal populations, optimizing their health and productivity [18]. In exploring the intestinal microbiota, metagenomics is an indispensable technology providing detailed insights into microbial communities. The 16S rRNA gene serves as a pivotal molecular marker for investigating the ruminal microbiota, offering insights into microbial community structure, taxonomy, and diversity. Alpha and beta diversity, which reflect species variability within and between communities, respectively, are key indicators in the study of the microbiome. These indices summarize the complexity and structure of microbial communities, providing information on the stability and function of the microbiome [22,23]. On the other hand, linear discriminant effect size analysis (LEfSe) is a powerful statistical tool that identifies significant biomarkers between groups, facilitating the understanding of functional and taxonomic diversity in the microbiome [24]. These methods allow us to explore the causes and consequences of microbial biodiversity in the intestine, as well as its contribution to intestinal health and function.

In conclusion, this study focuses on the effects of age on the intestinal microbiota of cattle and its correlations with blood parameters in the bovine genetic core. This study highlights the significant impact of age on the diversity and composition of the microbiota and its connection with various blood parameters. These findings emphasize the importance of understanding how the intestinal microbiota dynamics change with age and their relationship with the metabolic health of cattle. Our objective is to investigate this relationship, hypothesizing that as age increases, the diversity of bacterial communities also increases and the composition of these communities varies significantly. This research will guide the development of personalized probiotic therapies for different life stages, optimizing the microbiota composition and improving cattle performance.

## 2. Materials and Methods

### 2.1. Animal and Sample Collection

A total of 21 fecal samples from Simmental breed cattle were collected from the Central Genetic Nucleus of Donoso Agricultural Research Station (EEA Donoso in Spanish), a government-owned herd where a genetic nucleus of cattle is established, located in Huaral, Lima (128 masl; 11°31′18″ S and 77°14′06″ W). The conditions included an average temperature of 25.5 °C, a relative humidity of 88.5%, and exposure to natural light [25]. A preliminary pilot study was conducted to determine the optimal number of replications required for this research. The age groups were specified as follows: 4y5m corresponded to 58 to 63 months, 1y5m corresponded to 18 to 21 months, and 5m corresponded to 5 months. In all age groups, the sex ratio was 4 females to 3 males. The diet of all cattle included the same components, adjusted in different proportions according to the specific needs of each life stage. The feeding regime at the Donoso Experimental Station of INIA (Peru) is based on green forage, with specific supplements according to the age category. The 5m calves received 6 L of milk daily, divided into two feedings, along with 3.5% dry matter (DM) of body weight (corn silage) and 1.0 kg of balanced feed containing 17% crude protein (CP). Cattle from 1y5m were fed with 3.0% DM of body weight (corn silage) and 2.0 kg of balanced feed with 14% CP. Donors 4y5m received 1.4% DM of body weight (corn silage) and 2.0 kg of balanced feed with 18% CP. The corn silage contained 24–26% DM, corresponding to 74–76% moisture, and the feed had 90% DM, equivalent to 10% moisture. Fecal samples were collected directly from the rectum of each animal using disposable gloves, transported to the laboratory in liquid nitrogen, and stored at −80 °C until DNA extraction. Also, blood samples were collected from the jugular vein of each animal. The 21 cattle included in this study were part of the Central Genetic Nucleus. These animals are continuously monitored by the veterinary unit of the Donoso Genetic Center in Huaral to ensure they are healthy. Routine veterinary examinations, which include physical inspections, clinical history reviews, and laboratory tests, are conducted to maintain the high health standards required for semen and ovum donors. Consequently, there are no diseased animals in the genetic nucleus. This study was conducted by following the Peruvian National Law No. 30407: “Animal Protection and Welfare”.

### 2.2. Blood Parameters

Three mL of whole blood was collected in two BD Vacutainer^®^ K2 EDTA tubes (Becton, Dickinson and Company, Franklin Lakes, NJ, USA) and stored at 4 °C, while 4 mL of blood was allowed to clot at room temperature in BD Vacutainer^®^ SST tubes (Becton, Dickinson and Company, Franklin Lakes, NJ, USA) before being promptly transported to the laboratory. Both the SST tube and one K2 EDTA tube were centrifuged at 3000× *g* for 30 min to separate serum and plasma, respectively. The complete blood count (CBC) was performed using a Procyte Dx^®^ hematology analyzer (IDEXX Laboratories, Westbrook, MA, USA) with blood collected in EDTA tubes. The profiles of the test consisted of three types: red cell series and white cell series and platelets (PLT) (Table S1). The parameters of the red cell series include hematocrit (HCT), hemoglobin (HGB), erythrocytes (RBC), mean corpuscular volume (MCV), mean corpuscular hemoglobin (MCH), and mean corpuscular hemoglobin concentration (MCHC). The white cell series includes leukocytes (WBC), neutrophils (NEU), segmented (SEG), lymphocytes (LYM), monocytes (MONs), eosinophils (EOS), basophils (BAS), neutrophils (%) (NEU%), lymphocytes (%) (LYM%), monocytes (%) (MON%), eosinophils (%) (EOS%), and basophils (%) (BAS%) [26]. Plasma samples were used for biochemical analyses, which were carried out using a Bayer Advia^®^ 1200 chemical system (Siemens Medical Solutions Diagnostics, Tarrytown, NY, USA). Plasma was analyzed to determine total proteins (TP) and triglycerides (TG).

### 2.3. DNA Extraction and 16S rRNA Gene Sequencing

Genomic DNA was obtained from 21 fecal samples using the Stool DNA Isolation Kit (Norgen, Biotek Corporation, Sacramento, CA, USA), following the guidelines provided by the manufacturer. The concentration of the extracted DNA was measured using the NanoDrop ND-1000 spectrophotometer, and the absorbance ratio of 260/280 was determined to assess its quality. DNA integrity was checked through 1% agarose gel electrophoresis. For constructing the Illumina amplicon sequencing library, approximately 10 ng of DNA from each sample was subjected to PCR amplification using the 515F/806R primer pair for the 16S rRNA gene. The PCR protocol involved an initial denaturation at 98 °C for 1 min, followed by 30 cycles of denaturation at 98 °C for 10 s, annealing at 50 °C for 30 s, and elongation at 72 °C for 30 s, with a final extension at 72 °C for 5 min. Sequencing libraries were prepared using the Illumina TruSeq DNA PCR-Free Library Preparation Kit (Illumina, San Diego, CA, USA) according to the manufacturer’s instructions, including the addition of index sequences. Subsequently, the quality of the libraries was evaluated using a Qubit 2.0 Fluorometer (Thermo Scientific, Waltham, MA, USA). Finally, the validated libraries were sequenced using the 250 bp paired-end Illumina NovaSeq 6000 (Illumina Inc., San Diego, CA, USA) platform according to the manufacturer’s instructions.

### 2.4. Taxonomic Classification and Bioinformatic Analyses

A precise evaluation of sequencing reads was conducted using the FASTQC [27] and MULTIQC [28] tools. Subsequently, paired-end sequencing reads, previously demultiplexed by Illumina, were analyzed using the Qiime2 [29]-DADA2 [30] software v2023.9, resulting in the generation of an Amplicon Sequence Variants (ASVs) table. In order to minimize the chance of false positive ASVs, any distinct sequences with a combined abundance of less than 10 reads across all samples were excluded. Taxonomy assignment for the ASVs was performed using SILVA v138.1 for analyzing 16S sequences. The ASV tables underwent filtration to eliminate unidentifiable and undesirable phyla (such as cyanobacteria/chloroplasts) within bacteria. Specifically, the cyanobacteria/chloroplasts sequences were removed. Subsequently, the high-quality filtered sequences were aligned using the integrated MAFFT aligner [31]. Rooted and unrooted 16S phylogenetic trees were then built using the QIIME2 phylogenetic module, employing the FastTree algorithm.

### 2.5. Statistic Analysis

Statistical analysis of the data was performed using the R package Phyloseq [32] and Microeco [33] in R (v4.1.1) [34]. Rarefaction curves were produced for individual samples to evaluate the depth of sequencing. Subsequently, several intestinal bacterial alpha diversity measures were also assessed (Fisher, Faith’s phylogenetic diversity, ACE, Observed ASVs, Chao1, Shannon). A two-way ANOVA was employed to evaluate the effect of age and sex on these metrics and blood parameters. Beta diversity was assessed utilizing the Jaccard and unweighted Unifrac methods, and the results were visualized using Principal Coordinate Analysis (PCoA). Variations in bacterial communities across groups were assessed using two-way PERMANOVA [35] analysis, with 9999 permutations utilized for the evaluation. Distinctive features of gut microbiota profiles were identified using linear discriminant analysis (LDA) effect size with LEfSe [36]. Through LEfSe, we highlighted the biomarkers with the highest statistical and biological significance. The identified microbial biomarkers with statistical significance were reported with a *p*-value < 0.01. Spearman rank correlation analyses were conducted on variable pairs, encompassing blood parameters and bacterial alpha diversity indices and visualized with heatmaps in R. Also, the correlation between blood variables and soil bacterial community composition were analyzed by Mantel tests (999 permutations). Microbiota functions were predicted using the Functional Annotation of Prokaryotic Taxa (FAPROTAX) [37]. To evaluate the differences between groups of functional diversity, the Kruskal–Wallis test was used, followed by Dunn’s multiple comparisons test.

## 3. Results

In the bacteria, 1,699,347 high-quality filtered reads were obtained. On average, 80,921 high-quality reads were recorded per sample, reaching a maximum of 114,704 and a minimum of 64,021 high-quality reads. This variability in the number of reads reflects the diversity and richness of the bacterial sample analyzed. The rarefaction curve was used to investigate variations in gut microbiota communities (Figure S1). This curve revealed the expected diversity within the sampled bacterial communities and highlighted the optimization of sampling. Therefore, the data set was considered appropriate for further analysis.

### 3.1. Influence of Age on the Diversity and Composition of the Bovine Microbiome

**Figure 1:**
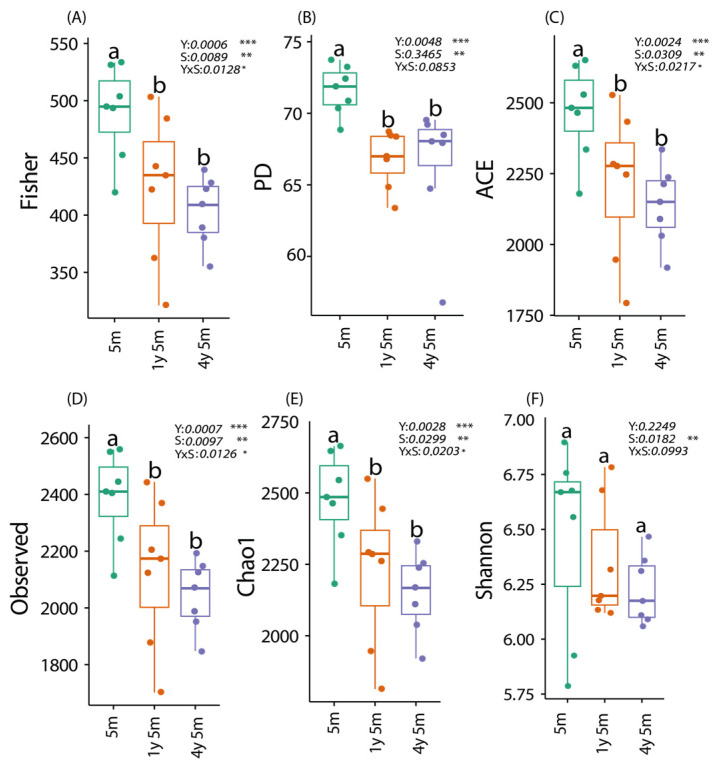
Alpha diversity metrics were evaluated in cattle in three age groups: 4y5m, 1y5m, and 5m. Diversity indices shown include Fisher, PD, ACE, Observed, Chao1, and Shannon (**A**–**F**). Each graph represents the distribution of diversity indices for both sexes within each age group. Statistically significant differences are indicated with different letters (a, b) according to Tukey’s post hoc tests. (* *p* < 0.05, ** *p* < 0.01, *** *p* < 0.001.)

A two-way ANOVA analysis of six alpha diversity indices (Fisher, PD, ACE, Observed, Chao1, and Shannon) revealed a significant influence of age (PD *p* = 0.0048; Observed *p* = 0.00073; Fisher *p* = 0.00063; Chao1 *p* = 0.0028; ACE *p* = 0.00245) and sex (Observed *p* = 0.00965; Fisher *p* = 0.0089; Shannon *p* = 0.018; Chao1 *p* = 0.003; ACE *p* = 0.03097), as well as their interaction (Observed *p* = 0.01261; Fisher *p* = 0.01279; Chao1 *p* = 0.02035; ACE *p* = 0.02166), on the alpha microbial diversity in cattle. However, for the Phylogenetic Diversity (PD) and Shannon indices, no significant effect of sex or the interaction between age and sex was observed (Figure 1).

The PD, Observed, and Fisher indices reflect the overall phylogenetic diversity, species richness, and abundance and rarity of species, respectively. On the other hand, Chao1 and ACE are species richness estimators that predict the total number of species in a community from sampling data. Tukey post hoc tests indicated that cattle of 4y5m exhibited significantly higher values in all indices compared to the other age ranges. The *p*-values presented in bold indicate statistical significance (*p* < 0.05). The results of the Tukey post hoc tests are represented by letters (a, b), denoting groups that differ significantly.

**Figure 2:**
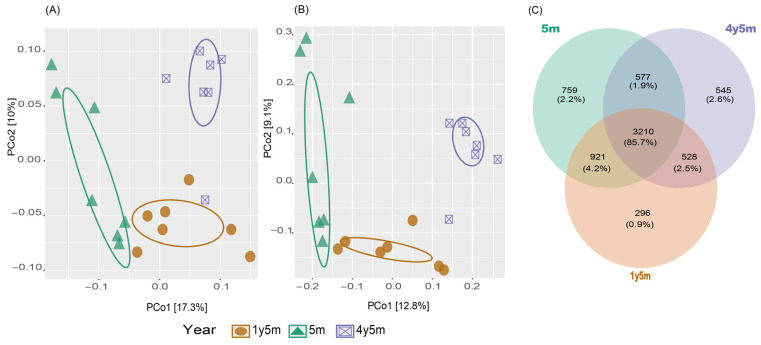
Beta diversity analysis and a Venn diagram illustrating the differences in microbial community composition among cattle of different age groups. (**A**) PCoA based on unweighted Unifrac. (**B**) PCoA based on Jaccard distance. (**C**) Venn diagram showing the shared and unique ASVs among age groups (4y5m, 1y5m, and 5m).

The congruence in the composition of the intestinal microbiota among the sets was determined through beta diversity, represented in Principal Coordinates Analysis (PCoA) graphs (Figure 2). The PCoA diagrams, based on unweighted Unifrac and Jaccard distances, indicated a significant variation in the composition of the bovine intestinal microbiota, differentiated by age ranges (Figure 2A,B). This variation was confirmed by the pseudo-F *p*-value in the pairwise PERMANOVA test, which turned out to be less than 0.05 when comparing all sets. Both metrics demonstrated statistical significance for the variables of age (unweighted Unifrac *p* = 0.013; Jaccard *p* = 0.001), age–gender interaction (unweighted Unifrac *p* = 0.012; Jaccard *p* = 0.009), and gender (Jaccard *p* = 0.011) (Table 1).

For both metrics, they revealed similar patterns in the distribution of microbial communities according to the different age ranges of the cattle. The results of the PCoA showed that the age groups 4y5m and 1y5m presented similarity in the composition of their microbial communities, evidenced by their proximity in the graphs. In contrast, cattle aged 5m formed a group clearly separated from the other two, indicating a different microbial composition in this age range (Figure 2A,B).

The illustrated Venn diagram highlights the unique and shared amplicon sequence variants (ASVs) among the three age groups of cattle: 4y5m, 1y5m, and 5m. The analysis revealed that the core microbiota consisted of 3210 ASVs, as shown in Figure 2C. The findings indicated the presence of 759 unique ASVs for the 4y5m group, 295 unique ASVs for the 1y5m group, and 545 unique ASVs for the 5m group.

### 3.2. Microbial Diversity among Bovine Bacteria

**Figure 3:**
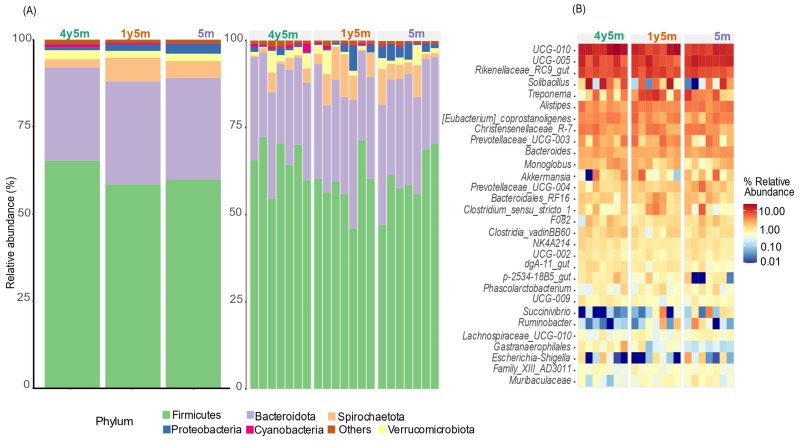
Relative abundance of phyla and genera in three different age groups of cattle. (**A**) Relative abundance of gut microbiota at the phylum level across different age groups (4y5m, 1y5m, and 5m). (**B**) Heatmap illustrates the relative abundance of the 30 most abundant bacterial genera among different age groups, highlighting variations in microbial community composition.

In fecal samples from all livestock groups, the dominant bacterial phyla were *Firmicutes*. In 4y5m, *Firmicutes* represented 60%, while in 1y5m they represented 58%, and in 5m they represented 65%. *Bacteroidota* were the second most abundant group, with 28% in 4y5m, 29% in 1y5m, and 26% in 5m. *Spirochaetota* were present at 4% in 4y5m, 7% in 1y5m, and 2% in 5m. Finally, *Verrucomicrobiota* represented 2% in 4y5m, 2% in 1y5m, and 2.5% in 5m (Figure 3A). 

The heatmap illustrates the relative abundance of various bacterial genera in different samples. The 30 most abundant genera were observed. The bacterial community results showed notable differences between the three age groups. In particular, the genera *UCG-010* and *UCG-005* exhibited high abundance in most samples, with *Rikenellaceae RC9* gut group standing out in the 4y5m group. *Treponema* was notably abundant in the 1y5m and 4y5m groups, while *Ruminobacter* and *Succinivibrio* also showed high abundance in the 1y5m and 5m samples, respectively. Other genera such as *Alistipes*, *[Eubacterium] coprostanoligenes* group, *Christensenellaceae R-7* group, *Prevotellaceae UCG-003*, and *Bacteroides* presented high abundance in different combinations of samples, especially in the groups of 4y5m, 1y5m, and 5m. On the contrary, the genera *Escherichia-Shigella*, *Succinivibrio*, *Ruminobacter*, *Muribaculaceae*, and *Akkermansia* showed low relative abundance in all samples analyzed. This analysis allows us to visualize differences in the bacterial composition between the samples, highlighting the variation in the presence and predominance of certain bacterial genera.

### 3.3. Biomarkers Identification and Correlation with Blood Parameters

**Figure 4:**
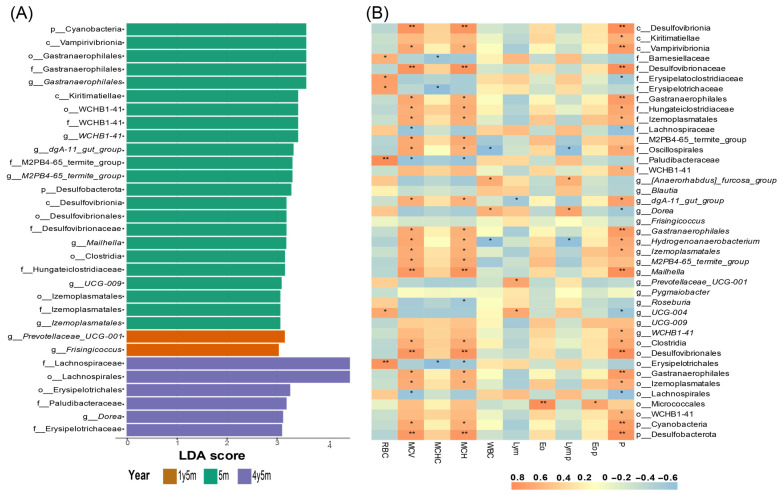
LEfSe analysis and Spearman correlation heatmap of biomarkers with blood parameters. (**A**) Linear discriminant effect size analysis (LEfSe) showing the most significant bacterial taxa differentiating between the age groups. The length of the bars indicates the effect size of each taxon. (**B**) Heatmap displaying Spearman correlation coefficients between identified bacterial biomarkers and significant blood parameters. Positive and negative correlations are represented by different colors (* *p* < 0.05, ** *p* < 0.01).

To determine specific bacterial taxa associated with different age categories, a comparison of fecal microbiota compositions was conducted. This analysis was performed using the linear discriminant analysis effect size method (LEfSe). The most significant differentiation in taxa, from phylum to genus level, was determined through an LDA score (Figure 4A). In the 4y5m group, the following taxa were detected, where twenty-three taxa were identified: two phyla (*Cyanobacteria*, *Desulfobacterota*), five orders (*Gastranaerophilales*, *WCHB1-41*, *Desulfovibrionales*, *Clostridia*, and *Izemoplasmatales*), three classes (*Vampirivibrionia*, *Kiritimatiellae*, and *Desulfovibrionia*), six families (*Gastranaerophilales*, *WCHB1-41*, *M2PB4-65_termite_group*, *Desulfovibrionaceae*, *Hungateiclostridiaceae*, and *Izemoplasmatales*), and seven genera (*Gastranaerophilales*, *WCHB1-41*, *dgA-11* gut group, *M2PB4-65* termite group, *Mailhella*, *UCG-009*, and *Izemoplasmatales*). In the group of 1y5m, two genera (*Prevotellaceae UCG-001* and *Frisingicoccus*) were identified. Finally, in the group of 5m, six taxa were found: two orders (*Lachnospirales* and *Erysipelotrichales*), three families (*Lachnospiraceae*, *Paludibacteraceae*, and *Erysipelotrichaceae*), and one genus (*Dorea*).

Spearman correlation analysis was employed to evaluate the relationship between biomarkers identified by LEfSe and hematological parameters (Figure 4B). Significant positive correlations were found between RBCs and six biomarkers, with r-values ranging from 0.6 to 0.8. Additionally, MCV and MCH consistently exhibited significant positive correlations with twenty biomarkers, with r-values between 0.6 and 0.8. Significant negative correlations were observed for MCV and MCH with one biomarker, showing r-values between 0.4 and 0.6. MCHC showed significant negative correlations with three biomarkers, with r-values ranging from 0.4 to 0.6. WBCs and LYMs% demonstrated significant positive correlations with two biomarkers (r-values 0.4 to 0.6) and significant negative correlations with two others (r-values 0.4 to 0.6). Eosinophils and eosinophils(%) exhibited significant positive correlations with one biomarker, with r-values ranging from 0.6 to 0.8. Finally, lymphocytes showed significant positive correlations with two biomarkers (r-values 0.6 to 0.8) and a significant negative correlation with one biomarker (r-values 0.4 to 0.6). Regarding TPs, significant positive correlations were observed with twenty-two biomarkers (r-values 0.6 to 0.8) and significant negative correlations with five biomarkers (r-values 0.4 to 0.6).

### 3.4. Relationship of Alpha/Beta Diversity with Blood Parameters

With the blood parameters (Table S2), a two-way ANOVA was performed considering age and sex. The significant parameters for age were the following: RBCs, MCV, MCH, WBCs, NEUs%, SEG%, LYMs%, NEUs, SEG, LYMs, and TPs. For sex, the significant parameters were the following: WBCs, NEUs%, SEG%, LYMs%, NEUs, SEG, and TGs. The interaction between age and sex was significant for WBCs, NEUs, SEG, and LYMs (Table S3).

**Figure 5:**
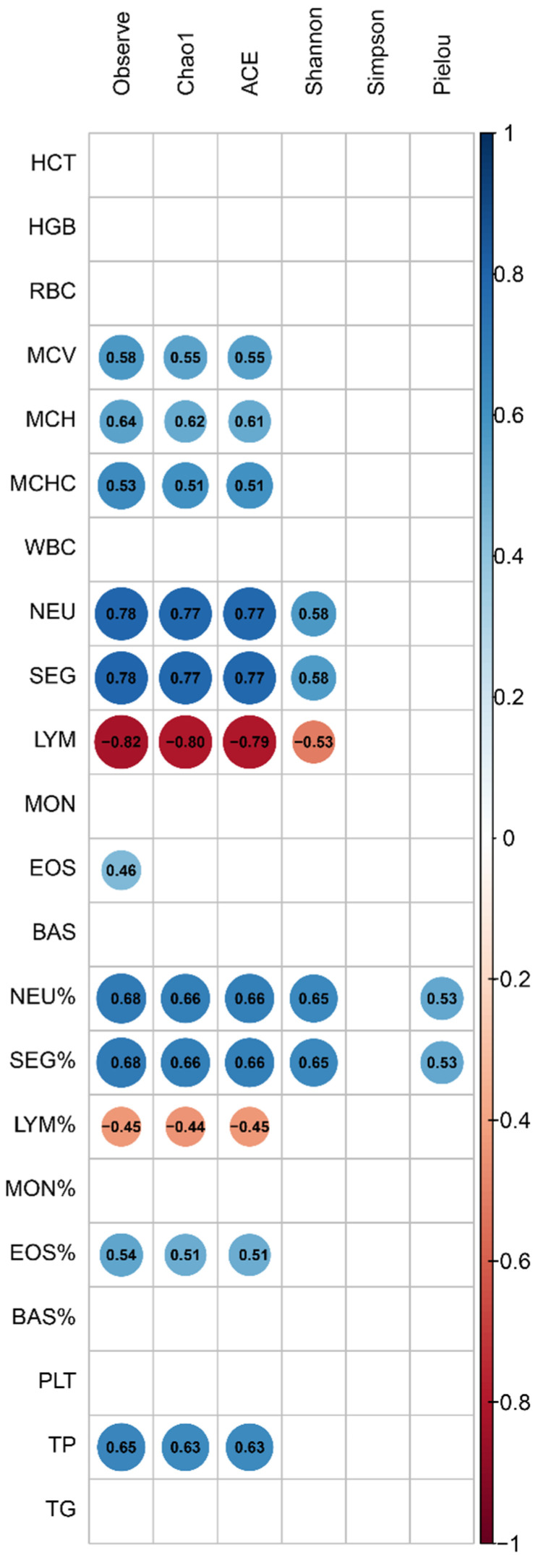
Spearman correlation between hematological parameters and alpha diversity. Positive correlations are represented in blue, while negative correlations are shown in red. Only significant correlations (with *p*-values < 0.05), are shown in the matrix, providing a clear view of the key interactions between blood parameters and microbial diversity.

A Spearman correlation analysis was conducted between blood parameters and alpha diversity indices (Figure 5). The Pielou index correlated positively with NEUs% and SEG% (r = 0.53). The Shannon index showed positive correlations with NEUs, SEG, NEUs%, and SEG% (r = 0.58–0.63) and a negative correlation with LYMs% (r = −0.53). Chao1, Observed, and ACE indices had significant positive correlations with EOSs, EOSs%, MCV, TPs, and MCH (r = 0.44–0.94), and negative correlations with LYMs% and LYMs (r = −0.44 to −0.63). 

In the correlation analysis of blood variables with beta diversity (Table S4), several significant results were found. For the Jaccard index in the Mantel test, RBCs showed a significant correlation (*p* = 0.004), as did MCV (*p* = 0.001), MCH (*p* = 0.001), WBCs (*p* = 0.035), and TPs (*p* = 0.001). In the Partial Mantel test, significant correlations were observed for RBCs (*p* = 0.016), MCV (*p* = 0.002), MCH (*p* = 0.001), and TPs (*p* = 0.001).

For the unweighted Unifrac index in the Mantel test, significant correlations were found for MCV (*p* = 0.002), MCH (*p* = 0.001), WBCs (*p* = 0.026), LYMs (*p* = 0.023), NEUs% (*p* = 0.038), and SEG% (*p* = 0.039). In the Partial Mantel test, significant correlations were also found for MCV (*p* = 0.017), MCH (*p* = 0.003), and TPs (*p* = 0.005).

### 3.5. Functional Diversity

**Figure 6:**
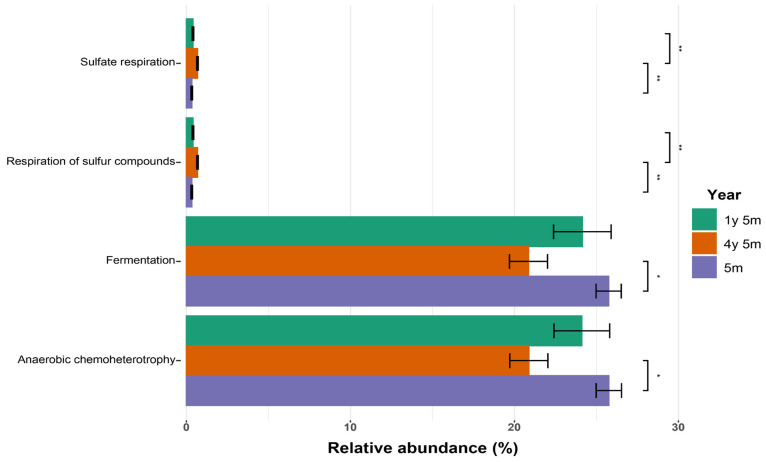
Relative abundance of metabolic functions in cattle of different ages. Relative abundance of metabolic functions in the intestinal microbiota of cattle from three age groups: 1y5m, 4y5m, and 5m (* *p* < 0.05, ** *p* < 0.01.)

Functional profiles of the microbiota, predicted using FAPROTAX, revealed significant differences between age groups (Figure 6).

Sulfate respiration was more abundant in the 4y5m group, showing a significant difference compared to the 1y5m groups. Similarly, respiration of sulfur compounds was also more abundant in the 4y5m group, with significant differences compared to the other two groups. In contrast, the 5m group exhibited higher abundance of fermentation and anaerobic chemoheterotrophy, with significant differences compared to the 4y5m group.

## 4. Discussion

The gut microbiota in ruminants forms a complex and interactive network that is crucial for metabolism, immune system regulation, nutrient absorption, and maintaining the integrity of the intestinal mucosal barrier [38,39]. Recent research has revealed that the gut microbiota in ruminants undergoes continuous changes and is affected by a variety of factors, such as dietary habits, species differences, and external environmental conditions [40,41]. In this study, we examined the bacterial diversity and abundance of the intestinal microbiota in Simmental cattle, taking into account variations in age and sex. Our findings revealed that older cattle exhibited significantly higher bacterial diversity and abundance compared to younger cattle, highlighting notable age-related differences in microbial composition.

Alpha diversity indices showed significant changes with age, specifically Fisher, PD, ACE, Observed, and Chao1 indices, reflecting variations in microbial diversity associated with aging. These findings are consistent with previous studies that reported an increase in alpha diversity with age in calves during the first 8 weeks of life [42], in cattle [43], and in goats [44]. These changes are attributed to factors such as the transition from a milk-based diet to a solid diet, immune system development, and gastrointestinal tract maturation [43]. As the gastrointestinal tract matures, it provides a more stable and diverse environment that supports a richer microbial community [40]. Additionally, the development of the immune system helps to regulate and maintain a balanced microbiota, allowing beneficial microbes to thrive and outcompete pathogenic species [39]. This maturation process not only alters the physical and chemical environment of the gut but also enhances the host’s ability to digest a wider variety of nutrients, further promoting microbial diversity [45].

Sex significantly influenced the alpha diversity indices Observed, Fisher, Shannon, Chao1, and ACE, showing differences in microbial composition between males and females, influenced by hormonal levels [46,47,48,49]. The interaction between sex and age was also significant, suggesting that these combined factors considerably impact microbial structure [50,51]. Differences in feeding behavior and physiology between sexes at different ages can influence gut microbiota diversity [11]. Estrogens can directly modulate the metabolism of the microbiota through the estrogen receptor beta and are major regulators of circulating estrogen, impacting microbial diversity and composition [52]. Additionally, testosterone has been shown to significantly affect the intestinal microbiota composition, with higher fecal testosterone levels associated with increased microbial diversity and distinct microbial community structures. Testosterone can inhibit the growth of certain pathogens, promoting a healthier gut microbiota [53].

In this study, beta diversity analysis showed significant variations in the intestinal microbiota across different age groups, with both age and the interaction of age and gender influencing microbiota composition. These findings align with previous research, highlighting the importance of age and gender in microbiota studies [44,54,55]. Age-related changes in the symbiotic microbiota of cattle, particularly in the ruminal microbiota at different developmental stages, are well-documented [5,54,56]. For instance, in yaks, the ruminal microbiota from birth to 12 years shows distinct age-related variations and maturation [57]. Studies on bovines and caprines have also highlighted notable alterations in rumen microbial diversity with age, both in cattle [55] and goats [44]. Additionally, age affects microbiota composition in cattle [58] and pigs [59], with significant interactions between age and gender observed in cattle [54] and goats [44]. The three age groups shared similar dominant bacterial phyla, including *Firmicutes*, *Bacteroidota*, *Spirochaetota*, and *Verrucomicrobiota*. These findings align with previous studies on cattle and other ruminants, such as goats [44,60], alpacas [61,62], and cattle [5,56].

The observed rise in butyrate-producing bacteria, particularly *UCG-010* and *UCG-005* in the 4y5m and 1y5m groups, indicates their potential contribution to butyrate production [59]. This increase underscores their role in enhancing butyrate levels, which are crucial for intestinal health. This, in turn, improves food digestion efficiency and promotes the consumption capacity and maturation of the intestine during weaning [63]. These bacteria are capable of converting lactate into acetate, propionate, and butyrate, which provide energy to the host, enhance intestinal barrier function, and reduce inflammation [64].

This study observed a gradual increase in the proportion of *Rikenellaceae RC9* in the 4y5m and 1y5m groups, which are crucial for fibrous plant degradation, as demonstrated by metagenomic [65] and transcriptome analysis [66]. These bacteria contribute to breaking down polysaccharides such as starch, cellulose, and lignin in the hindgut [67,68,69]. This increase suggests an adaptation of the gut microbiota to more efficiently degrade complex carbohydrates as cattle age. On the contrary, the genus *Escherichia-Shigella* was found in low abundance, which coincides with research linking it to diarrheal diseases [70,71]. This low abundance suggests that the absence of significant levels of *Escherichia-Shigella* indicates a lower incidence of diarrhea in our study population, as *Escherichia-Shigella* thrives in compromised intestinal conditions that cause inflammation and diarrheal symptoms [72,73]. Therefore, the limited presence of this genus in our findings may reflect a generally healthy gut microbiota in the cattle sampled, further supporting the notion that the health status of the animals plays a significant role in shaping their microbial communities.

A low abundance of the fermentative bacteria *Succinivibrio* and *Ruminobacter* was observed in the three age groups of cattle, with a most notable decrease in the 5m group. This finding aligns with the study that reported low proportions of *Succinivibrio* and *Ruminobacter* in cattle, especially in comparison with dominant genera such as *Prevotella* [74]. The low abundance of these genera may reflect normal variations in the microbiota associated with age and adaptation of the microbiome to different stages of development [55,75]. Furthermore, other bacterial genera such as *Bacteroides* can take on similar roles in carbohydrate fermentation, thus ensuring digestive efficiency [76]. *Bacteroides* showed a higher proportion in this study, underlining their importance in the degradation of a wide variety of carbohydrates, which is essential to maintain digestive function and produce volatile fatty acids necessary for the energy metabolism of ruminants [77,78].

*Mailhella* was identified in the group of 4y5m cattle, consistent with previous studies that also reported in cattle [79]. The presence of *Mailhella* may indicate an adaptive microbiome that helps maintain intestinal balance, especially in situations of inflammation or stress [80,81]. *Frisingicoccus*, identified as a biomarker in the 1y5m group, suggests a possible protective role in intestinal health by contributing to the reduction of harmful compounds such as NH3-N and improving nutrient digestion and absorption [82,83]. Furthermore, in the group of 5m, the genus *Dorea* was identified, known for its ability to regulate intestinal health and nutrient absorption [84].

The genus *Dorea* showed significant positive correlations with leukocytes and lymphocytes, suggesting its role in modulating immune health and intestinal function by facilitating interactions between epithelial and immune cells [85]. *Prevotellaceae UCG-001* also presented a positive correlation with lymphocytes, supporting its role in enhancing immune response and homeostasis [86]. Additionally, Lachnospirales had a significant negative correlation with proteins, indicating its influence on protein metabolism and nutrient absorption, aligning with studies linking its abundance to metabolic and inflammatory disorders [87,88]. *Roseburia* is a butyrate-producing bacteria that helps prevent intestinal inflammation and maintain energy homeostasis [89]. A significant negative correlation has been found between *Roseburia* and MCH, suggesting that alterations in its abundance could affect metabolism and systemic health.

The observation of a significant positive correlation between the alpha diversity of the gut microbiota and neutrophil levels in the studied subjects aligns with previous research suggesting that the gut microbiota plays a crucial role in regulating the innate immune system, including the production and function of neutrophils [90]. The gut microbiota produces various microbial components and metabolites that influence hematopoiesis and neutrophil maturation [90]. Higher microbial diversity provides a broader range of beneficial signals that promote efficient neutrophil function, consistent with studies showing that microbial diversity is associated with better immune system regulation and the prevention of chronic inflammation [91]. Similarly, the significant negative correlation between the alpha diversity of the gut microbiota and lymphocyte levels observed in our study is consistent with evidence suggesting that lower microbial diversity is associated with immunological alterations [92]. These findings underscore the potential importance of the gut microbiota in influencing the immune response. Further research is needed to explore how these correlations might inform our understanding of immune-related conditions.

A higher prevalence of anaerobic chemoheterotrophy occurred in cattle aged 5m, which agrees with previous research that shows variations in the microbial composition of the rumen between various breeds of sheep, where processes such as fermentation and anaerobic chemoheterotrophy are constant, despite differences in microbial diversity [93]. Anaerobic chemoheterotrophy, essential for the digestion of plant fiber and the generation of volatile fatty acids, tends to be more active in young cattle, probably due to their high energy needs and the active development of the rumen [94].

This study does not include comprehensive metabolic analyses correlating microbiota changes with specific age-related variations. Further research is necessary to explore the metabolic pathways and their interactions with the microbiota at different ages. These considerations suggest important areas for future research to better understand the complex interactions between microbiota, age, and reproductive status.

## 5. Conclusions

This study examined the intestinal microbiota of the genetic nucleus of cattle, focusing on the impact of age and sex on its diversity and composition. Older cattle exhibited greater bacterial diversity and abundance, while sex significantly influenced microbial composition. In particular, increases in beneficial bacteria, such as butyrate-producing bacteria, were observed, which are crucial for intestinal health and reducing inflammation. Variations in the microbiota were also correlated with changes in blood parameters, highlighting the relationship between microbial diversity and the overall health of the cattle. The diet of all cattle consisted of the same ingredients, adjusted in different proportions to meet the specific needs of each life stage. These findings suggest that future strategies could focus on manipulating the microbiota to improve cattle health and performance. Specialized diets and probiotics tailored to different ages and sexes could be developed to optimize microbial composition and overall health.

## Figures and Tables

**Table 1:** PERMANOVA of unweighted Unifrac and Jaccard methods * *p* < 0.05, ** *p* < 0.01.

	Items	Df	SumOfSqs	R2	F	*p*-Value
Unweighted Unifrac	Year	2	0.10321	0.19044	2.45645	0.013 *
	Sex	1	0.02385	0.04401	1.13542	0.323
	Year:Sex	2	0.09975	0.18406	2.37413	0.012 *
	Residual	15	0.31512	0.58147		
	Total	20	0.54194	1		
Jaccard	Year	2	0.79733	0.18519	2.18551	0.001 **
	Sex	1	0.27793	0.06455	1.52362	0.011 *
	Year:Sex	2	0.49401	0.11473	1.35408	0.009 **
	Residual	15	2.73621	0.63551		
	Total	20	4.30549	1		

## Data Availability

The raw data supporting the conclusions of this article will be made available by the authors on request.

## Supplementary Materials

The following supporting information can be downloaded at https://www.mdpi.com/article/10.3390/microorganisms12071331/s1. Figure S1: Rarefaction curves of species richness show the sequencing depth of 16S data obtained from gut samples. Table S1: List of abbreviations. Table S2: Blood parameters of three age groups. Table S3: Two-way ANOVA results for blood parameters in cattle by age and sex. Table S4: Correlation of blood parameters with beta diversity (Jaccard and unweighted Unifrac) using Mantel and Partial Mantel tests.
