# Supplementary material for: Effects of Age in Fecal Microbiota and Correlations with Blood Parameters in Genetic Nucleus of Cattle
Source: Microorganisms. 2024 Jun 29;12(7):1331. doi: 10.3390/microorganisms12071331 (PMC11279168; doi:10.3390/microorganisms12071331)
Supplement: Supplementary file 1 [file microorganisms-12-01331-s001.zip › Supplementary Figure.pdf]

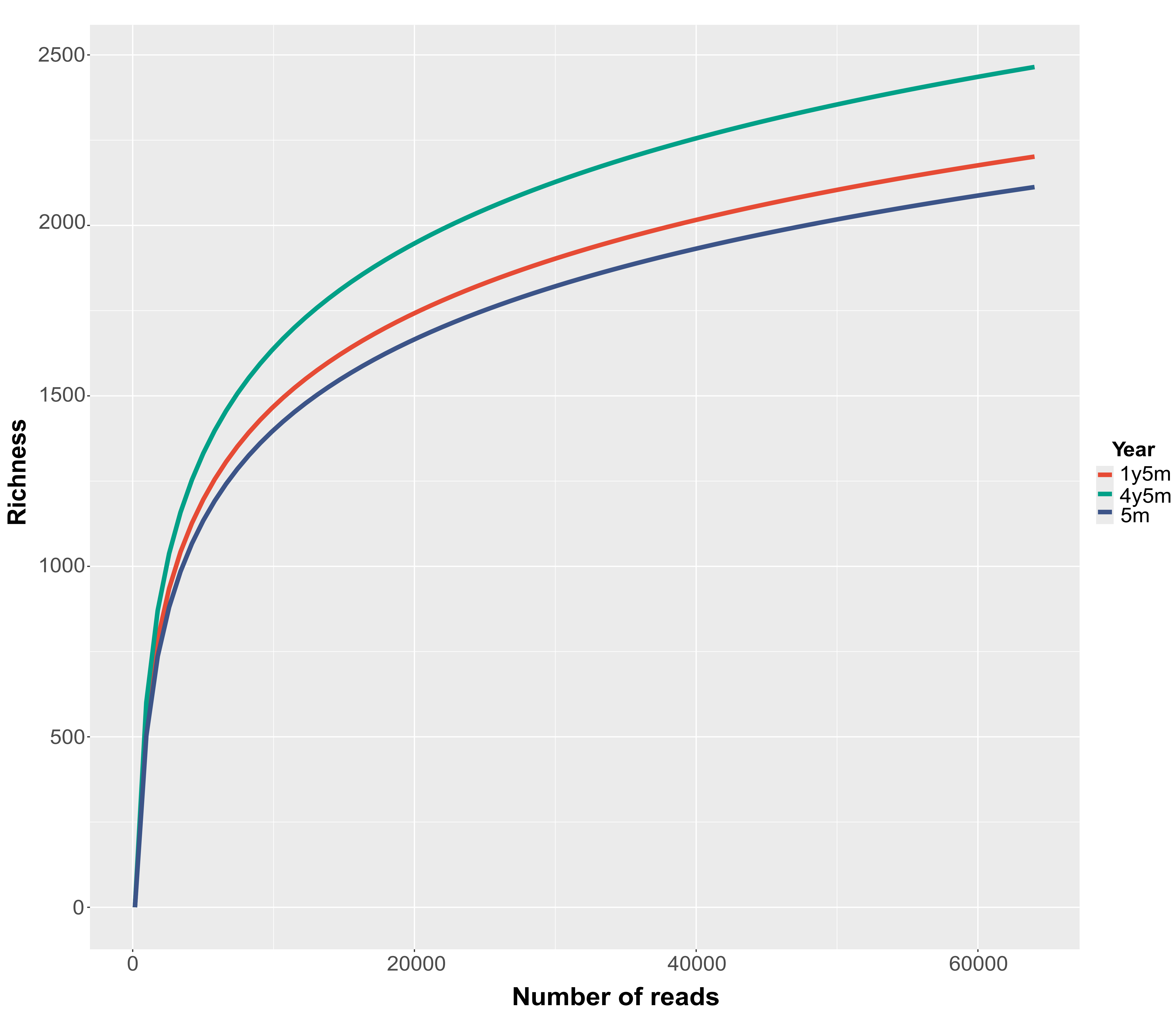

Figure S1: Rarefaction curves of species richness show the sequencing depth of 16S data obtained from gut samples
